# Mr. Yeatman in Answer to Mr. Bush's Remarks on His Reply to "an Old Practitioner."‡

**Published:** 1822-08

**Authors:** 


### Art. VI.—Mr. Yeatman *in Answer to* Mr. Bush's *Remarks on his Reply to* "*an Old Practitioner.*"‡

"Justum et tenacem propositi virum, &c."

1st. Mr. Bush has acknowledged himself to be the author of a communication, "on the Frauds practised by Persons to gain admission to the Medical Profession, and the Necessity of counteracting them; by an Old Practitioner."

The *Old Practitioner* should not exhibit his *little* productions in manuscript; for, although this may gratify an overweaning vanity, it may enable persons to judge of the proper application of the motto prefixed to his remarks:—"Qui odium in præcordiis fovet, viperam amplexuro similis est."

Mr. B. has not denied that I was the "practitioner" aimed at in his anonymous attack; although accused of this intention in so pointed a manner, (the *quo animo* attributed to me,) that he has been compelled to relinquish *bush-fighting* for *open* war; and by "the urchin, too, who has played the truant, and who betrays himself by trembling at the sight of the rod!"

The instances of irregular practitioners, noticed in the

‡ Mr. Bush has declared, in a note to the Editors, that he has "no wish to continue so unprofitable a subject;" and we recommend a similar line of conduct to Mr. Yeatman.—Without wishing to judge between the parties, we would remark, that the differences of individuals can have little interest for the public at large, and that the insertion of such discussions is entirely foreign to the objects of this Journal.—Edit.

Remarks, happened, as I am informed, in this town, some five-and-twenty years ago. My readers, therefore, will readily perceive that those instances could not have prompted Mr. B. to efforts which he appears to make in order to cleanse the Augean stye, a task requiring, alas! herculean strength;—and my *worthy friend* is no giant!

The following serious allegation occurs in Mr. B.'s anonymous communication:—"I live in the neighbourhood of a practitioner who has taken upon himself to qualify pupils for medical practice in the short space of a few months;" and in his Reply, instead of proving the allegation, he breaks out into open abuse.

2d. Mr. Bush alludes sarcastically to anatomical lectures in Frome, wishing, doubtless, that my pupils were only taught to prescribe for paupers, without knowing any thing of anatomy, or the principles of their profession: a great defect this, in that medical education (with reverence be it spoken,) which is derived in the country, and which I have conscientiously endeavoured to remedy. How I have discharged this duty, the progress made by my pupils will best prove. If I had expressed an opinion that anatomical lectures can be delivered in Frome with the same advantage to pupils as in London, where bodies are easily procured for dissection, I should have deserved censure; but, professing only (as my Remarks will show,) to *prepare* pupils for the great medical schools, as a clergyman prepares his for the universities, Mr. Bush's sarcasm loses its point.

3dly. Mr. B., in his anonymous attack, affirms, "that the law precludes all persons from admission into the chartered bodies of surgeons and apothecaries, but such as have *(bona fide)* served a regular term of five years as apprentices." The late editor, Dr. Hutchinson, having, in a note to Mr. B.'s communication, explained the law on these points, and having myself explained to him the rules of the College of Surgeons respecting candidates, he has chosen to shift his ground, rather than acknowledge his error; and now says, "the Apothecaries'" (not a word about surgeons,) "late Act renders it illegal for any person to practise as an apothecary, or an assistant, who has not served an apprenticeship of five years, and submitted to a subsequent course of study." "The pupils, therefore, he (Mr. Y.) may have taken for a less term than five years cannot become legally qualified for general practitioners, without some new arrangement. I would pardon delinquency arising from ignorance, but mark with severe censure any intentional evasion of the Act; and which, I think, Mr. Yeatman's reply shows he has been guilty of." Now, I would pardon delinquency arising from ignorance, but mark with severe censure any intentional evasion of the *argument*, when such an evasion is to illegalize my pupils. Mr. B. spoke of the law as it respects the admission of persons into the chartered body of surgeons, as well as apothecaries; and, without admitting his blunder respecting the former, he now only confines himself to the latter.

Since I have been in private practice, I have had, in all, two apprentices and two pupils: my youngest brother (M.D. member of the Royal Colleges of Surgeons in London and in Edinburgh, and of the Royal Medical Society of Edinburgh, in the Hon. the East India Company's service), two lieutenants of the Royal Navy, and the only son of a lady at Christchurch, Hants; all of whom are well known and much respected.—Are these the individuals who either *have* disgraced, or who mean to disgrace, themselves and me, by becoming practitioners without proceeding to the great schools of medicine? Are these the pupils of a surgeon, of whom my *worthy friend* says, "I live in the neighbourhood of a practitioner who has taken upon himself to *qualify* pupils for medical practice in the short space of a few months?"

Before I commenced the system of professional education, which I shall ever extend to my apprentices and pupils, I wrote to the Royal College of Surgeons a letter, to which I received the following reply, with a printed copy of the rules of the College respecting the admission of candidates for the honour of a surgeon's diploma.

"Sir,—By the other side you will perceive that an apprenticeship is not necessary to entitle a candidate to examination at this College; the requisition being, that he shall have been five years engaged in the acquisition of professional knowledge, of which the year's attendance at an hospital, mentioned on the other side, will, of course, count as one.

"I am, Sir, your most obedient servant,

"EDM. BELFOUR, Sec."

"*7th April*, 1820."

Again: "The present editor received a diploma of member of the Royal College of Surgeons, which enabled him to serve in the Royal Navy, without ever having been an apprentice, by simply exhibiting, on the day of examination, the usual certificates of having received a regular medical education.—A. B. G."—*See* Dr. GRANVILLE's *note to the second page of my Reply*.

It follows, therefore, that Mr. B. has betrayed a want of common information on this subject. Should he, however, be of opinion that mere parchment, binding an individual to serve five years as an apprentice, will make a youth wiser or more regular in his education, than certificates proving that he has been five years "engaged in the acquisition of professional knowledge," Mr. B. had better become *dictator* to the Royal College of Surgeons, which title, adorned with appropriate emblems, may be engraven, framed, and hung up in his sitting-room, by the side of Dr. Walker's diploma of vaccinator to the London Vaccine Institution.

4thly. My *worthy friend and neighbour* confesses that, "at a meeting of the whole of the medical practitioners of Frome," he was induced to hazard "observations" questioning the propriety of amputation in the case of Sarah Slade, although he never saw her. Now, this case was one in which complete amputation at the instep was performed; the first of the kind, after the manner of Chopart, performed in Great Britain, with the exception of those of the late Mr. Hey, of Leeds; and this I was not aware of at the time.

The remarks with which Mr. B. has now thought proper to *enlighten* the medical world, are really very amusing. He notices "an error in the first date of some importance," which is obviously a *misprint;* and he is the first to discover that the child had been subject to *chilblains* up to the 25th of March, for which, he says, she was under the care of a surgeon. Unfortunately, however, for Mr. B.'s veracity, the surgeon (I am told) to whom he has alluded, denies this; and the parents have just positively affirmed, that the child had not been subject to chilblains for a year and a half before the mortification came on! But, supposing chilblains had occurred, about the 25th of March, to a child four years old, living upon a dirt floor and in a damp situation, and just recovered from low fever, what has this to do with that weakened vitality of the parts, (unless, perhaps, to increase it,) which developed itself in the manner described in the 36th volume of this Journal? Mr. B. has thought proper to quote my words of the 28th of March in the following way:—"Mr. Yeatman's words are, 'a small portion of the foot appeared to be affected' only;" and jesuitically insinuates that, under such circumstances alone, I thought it necessary to remove all that portion of the foot anterior to the tarsal bones: whereas, on referring to the case, it will be clearly seen that this was done some time after the line of separation between the dead and living parts had (on the 30th,) formed around the foot *at the middle of the metatarsal bones;* and (on the 2d of April,) the sloughs had bared the *anterior half of the metatarsus*, and the whole of the first phalanges!

Had Sarah Slade been under Mr. Bush's care, and he had left nature to have formed her own stump, all the toes would have separated at their junction with the metatarsus; and this stump, after passing through the tedious processes of suppuration, granulation, and cicatrization, would have been left in a weak and mutilated state, without a cushion, subject to sinuses, liable to ulcerate (as all newly-formed parts are,) on receiving the least injury, and to frequent exfoliations from the black and denuded portions of the five metatarsal bones.—See vol. 37 of this Journal, in which an engraving of the amputated foot at the instep is given.

"Parts will often separate without amputation; but, if we have reason to suspect that the stump will be very long and tedious, or that it would exhaust the patient in the efforts to repair so extensive an injury, it will be preferable to take off the limb. If the mortification have been considerable, the cure would at least prove troublesome, the stump of a bad form, and ultimately of little utility to the patient."—Sir A. COOPER's *Lectures on Surgery*, according to SYDER's edition.

In allusion to a minor circumstance in Slade's case, Mr. B. says, "I think" (I wish he would *think*,) "a little attention to Mr. Yeatman's sequel will show that he was mistaken, and that it was not a black and denuded point of os calcis which he saw: every surgeon ought to know that a bone, when denuded and black, cannot furnish granulations till exfoliation has detached that portion:" and, thinking me "mistaken in the state of the *heel*," very *logically infers* that I was mistaken" "as to the state of the *toes* also." Now, *every surgeon ought to know* that a black and denuded *point* of os calcis,"—*not* "*a bone*," *point* or *speck of bone*,—may come away in the poultices or dressings, so as not to be perceived.

"The surface of bone which the instruments touch," (alluding to operations on bone,) "is, I believe, generally killed, but not to any considerable depth: instead, therefore, of coming away as a whole, the dead bone comes away in very thin scales with the dressings; this has been called insensible exfoliation."—Professor WILSON, in one of his Lectures delivered before the College; see his late work on the *Bones and Joints*, p. 287.

A black and denuded point or speck may be sometimes seen, both in the flat bones and those of a more spongy and vascular kind, as well after a small slough ("of the size of a shilling") has taken place of the superincumbent soft parts, as after operations on bone, without exfoliation having subsequently happened, so as to be perceived, or in any way to retard the healing process. Indeed, for aught that is known to the contrary, a point or speck of bone, though denuded and black, may be removed *by absorption*, and may not even pass off in the dressings, much less exfoliate, in the common acceptation of the term.

Mr. B. blames me for not calling in a second opinion in the case of this little pauper. Could there have been a reasonable doubt entertained on the propriety of the operation, the patient should most certainly have had the advantage of a second opinion, besides the assistance of my brother, who, at that time, was with me, in the last year of his apprenticeship. The child, it seems, is peculiarly fortunate in not having had the benefit of Mr. Bush's opinion.

My *worthy friend* speaks of the nail of my fore-finger, with which I moved the slough on the heel a little aside, the better to ascertain the true state of the bone, as "a clumsy substitute for a probe." Perhaps he will indulge the medical public with a graphic sketch, and an accurate description, of a rough piece of iron, (an *injurious* as well as "a *clumsy substitute*" for a pasteboard, bandage, &c.) which he desired a blacksmith to make, while a man, with fractured jaw, stood trembling at the anvil, to receive the benefit of his discovery. This patient soon fell under my care, and the fracture was treated successfully in a less *Cyclopean* manner.

> "Ah me! what" troubles "do environ
> The man that meddles with cold iron."

5thly. If Mr. B. employed sutures in a severely-contused lacerated wound of the thigh, in consequence of noticing the case "superficially," it is to be hoped that he will be less superficial and precipitate on a future occasion. I now find him, *prosequitur pavitans et ficto pectore fatur*:—"About six years ago, John Curtis, a nightly prowler, in his wanderings fell into a saw-pit, near my house. He was brought into my surgery in the morning, at a moment when I was passing through it to attend some urgent call. I noticed the case superficially, but saw there was a wound of considerable extent on the thigh. I had not the care of the poor; but ordered him to be taken care of, from humanity only:" and, after a *superficial* view of the patient, he gives a strong proof of his "*humanity*" in the following words:—"Mr. Cradock, who was then my apprentice, was requested to attach the edges of the wound by the interrupted suture!" and, lastly, adds, "and sent him to the workhouse, where he was under the care of Mr. Yeatman, who was parish-surgeon for that year. If inflammation came on, it was his duty to cut the sutures, and apply such topical and general remedies as the case required."

In my communication on the Application of Sutures to severely-contused lacerated Wounds, (which I transmitted for insertion before I heard of Mr. B.'s intention to publish any remarks on my Reply,) my *worthy friend* will perceive that mortification, in the case of Curtis, was the result of their improper use; although, the instant I saw the patient at the workhouse, I removed "the sutures, and applied such topical"

and administered "such general remedies, as the case required."

Mr. S. COOPER justly observes,—"In the present schools of surgery, the use of sutures is not recommended as it was in former days. In general, the promotion of union by the first intention cannot be set forth as a valid argument in favour of sutures being commonly used. Inflammation, above a very moderate pitch, always destroys every prospect of this nature, and occasions the secretion of pus instead of the exudation of coagulating lymph. Sutures have fallen into disrepute principally because they tend to increase inflammation. The new wounds which they make, their irritation as extraneous bodies, the forcible manner in which they drag the living parts together, and their incapacity, in general, to accomplish any useful purpose which position, adhesive plaster, and bandage, cannot effect, are strong motives for reprobating their being commonly used."

6thly and lastly. "The case of diseased stomach, to which Mr. Yeatman refers, is one of considerable interest: he can tell how he obtained the patient, what he supposed the case to be, and the medical treatment. I attended only the examination *post mortem*. However, I took minutes of the appearances, and may be able to correct any inaccuracies into which he might be led in that part of its history."

"He can tell how he obtained the patient;"—yes, and why it is my happiness to retain the confidence of the family of the deceased;—"what he supposed the case to be, and the medical treatment." On this head, two letters written to Dr. (now Sir GEORGE) GIBBES,—one a short time after I was called upon to attend the case alluded to, the other just after the patient's decease,—will, for the present, suffice.

"*Frome; December* 25, 1817.

"Sir,—The pil. hydrag. and the infus. cascarill. cum tinct. rhæi, have been employed in the case of Mr. ——, since I had the honour of seeing you on the 10th instant; but, finding the evacuations scanty, and very dark and offensive, I have occasionally had recourse to the extr. colocynth c. Two or three evacuations have, for several days past, resulted from the united effects of those remedies; but the last evacuation is as dark and offensive as the first.

The symptoms have been as follow:—Languor, nausea, vomiting,—acid eructations,—a quick and weak pulse, alternating with a languid pulse of 80, and a pulse intermitting at about every sixth stroke,—a dislike for food,—the conjunctiva slightly tinged,—the complexion sallow,—urine turbid, but not high,—no particular thirst,—the tongue slightly furred.

"This gentleman has been evidently losing flesh and declining in strength for some considerable time, and indeed has been always of a constitution radically delicate. He feels no particular pain on pressing in the epigastric or hypochondriac regions; but there is an evident fulness in the former, and he complains of a sense of fulness there. Perhaps, there is not only a sluggish state of the liver and a disordered condition of the digestives generally in this case, but an enlargement and induration of some of the viscera, not to be easily detected; which, with the impediment offered to the circulation in the vena portarum, may account for the intermitting pulse and most of the other symptoms.

"I find the application of a blister highly serviceable in allaying vomiting.

"I am, &c. &c."

"*To George Gibbes, M.D. Bath.*"

"*Frome; February* 17, 1818.

"Sir,—Since I had the honour of addressing you in the case of the late Mr. ——, very frequent vomitings, and extraordinary emaciation, have occurred, in addition to those symptoms before stated, and which led me to suspect, still more strongly, some organic affection of the pylorus and of the abdominal viscera.

"The friends of the patient being anxious to obtain professional aid on the spot, prevented my having that further assistance which your experience and talents could have afforded; but, as you are at all times alive to the interests of medicine, you may, very possibly, like to know the means latterly resorted to, and the appearances exhibited *post mortem*.

"Blisters to the epigastrium, the various internal remedies for allaying vomiting, the occasional exhibition of aperient injections, were, at different times, employed with little or no benefit. The patient rejected every thing the instant it was taken into the stomach. The daily immersion of the body in a tepid bath highly impregnated with meat, and the frequent use of mutton-broth injections, alone sustained the wreck of the constitution during the last month or six weeks. The last symptom was spasm of the bowels.

"About four or five weeks prior to the patient's decease, I discovered an organized substance in the situation of the pylorus; which was on the next day felt, though indistinctly, (in consequence of a more than usual fulness in the epigastric region,) by one of the medical gentlemen in attendance.

"*Dissection, 3d February*, 1818.—On exposing the abdominal viscera to view, a general appearance of disease is observable. Stomach: the external appearance of, tolerably natural. Cardiac orifice natural, except that the vessels of its internal coat are unusually filled with blood. A tumor, about the size of a walnut, presses on the outer and upper part of the pylorus, which, narrowing the outlet of the above organ, just admits the introduction of a large-sized quill. There is considerable ulceration both above and below the tumor internally; and a portion of the duodenum, which is ulcerated, is very much discoloured, and of so tender a structure as to be broken down by the fingers, while a ligature is applied previous to the removal of the stomach.

"Liver small, and the relative size of the right and left lobe very disproportionate; the latter being very small. The right lobe is of a much firmer texture than common; and, upon being cut into, exhibits a change of structure approaching to schirrus.

"Gall-bladder unusually distended with black and highly vitiated bile.

"Mesenteric glands diseased throughout; and, when divided, have a strumous appearance.

"The contents of the thorax quite natural.

"I am, Sir, truly and obediently yours,

"J. C. YEATMAN."

"*To George Gibbes, M.D. Bath.*"

*Frome, Somerset; July 4th,* 1822.

